# Femoral Nerve Injury After Prone-Position Lumbar Discectomy: A Case Report Highlighting the Role of Ultrasound in Recovery Monitoring

**DOI:** 10.7759/cureus.76653

**Published:** 2024-12-30

**Authors:** Mamoru Matsuo, Yoshitaka Nagashima, Yusuke Nishimura, Ryuta Saito

**Affiliations:** 1 Department of Neurosurgery, Nagoya Central Hospital, Nagoya, JPN; 2 Department of Neurosurgery, Nagoya University Graduate School of Medicine, Nagoya, JPN

**Keywords:** femoral nerve, nerve gliding, postoperative peripheral nerve injury, postoperative rehabilitation, prone-position surgery, rectus femoris, tinel-like sign, ultrasound

## Abstract

Postoperative peripheral nerve injuries are well-recognized complications of surgical positioning. In prone spinal surgeries, lateral femoral cutaneous nerve injuries are common, but femoral nerve injuries are rare. We present a case of femoral nerve injury following prone-position spinal surgery, highlighting the role of ultrasound imaging in diagnosis and management. A 79-year-old man developed anterior thigh pain, along with iliopsoas and quadriceps weakness, following lumbar discectomy at the L3/4 and L4/5 levels. Neurological evaluation revealed a positive Tinel-like sign over the rectus femoris near the inguinal ligament. Ultrasound showed no hematoma but suggested nerve traction or compression. Targeted rehabilitation significantly improved pain, muscle strength, and function within two weeks. Ultrasound imaging confirmed enhanced femoral nerve mobility, which correlated with symptom resolution. This case highlights the importance of distinguishing femoral nerve injuries from radiculopathy and demonstrates the utility of ultrasound for diagnosis and monitoring recovery. Although femoral nerve injuries in prone surgeries are uncommon, awareness and early rehabilitation are critical for favorable outcomes.

## Introduction

The femoral nerve, originating from the L2-L4 levels of the lumbar spine, traverses between the psoas major and iliopsoas muscles before passing beneath the inguinal ligament and continuing deep along the anterior thigh [1,2]. Injury to the femoral nerve may lead to deficits in knee extension and hip flexion, accompanied by sensory disturbances in the anterior thigh and medial aspects of the leg and foot [2]. Such injuries are recognized complications of various procedures, including iliopsoas, pelvic, and hip surgeries [1].

Postoperative peripheral nerve injuries related to surgical positioning are well-documented in clinical practice [1,3,4]. Among spinal surgeries performed in the prone position, lateral femoral cutaneous nerve injuries are the most commonly reported [5,6]. In contrast, femoral nerve injuries associated with prone-position spinal surgeries are exceptionally rare [1,7,8]. Consequently, standardized protocols for the prevention, diagnosis, and management of femoral nerve injuries in this context remain undeveloped.

This report describes a rare case of femoral nerve injury following spinal surgery performed in the prone position. It discusses preventive strategies, diagnostic considerations, and therapeutic interventions, including the utility of ultrasound imaging.

## Case presentation

**Figure 1:**
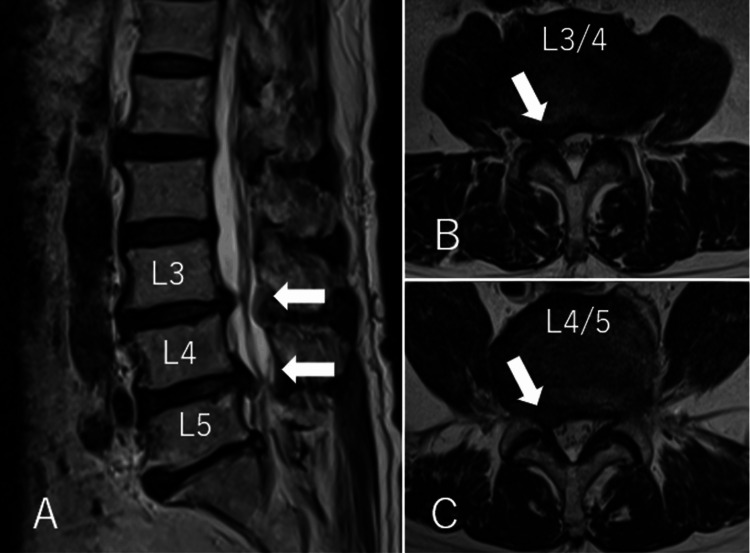
Preoperative MRI (A) Sagittal. (B, C) Axial. T2-weighted MRI performed preoperatively showing lumbar disc herniation (white arrow).

A 79-year-old man presented with pain in the lateral aspect of his right thigh. The patient was 171 cm tall, weighed 63.6 kg, and had a body mass index (BMI) of 21.7. Neurological examination revealed no significant muscle weakness in either lower limb. Magnetic resonance imaging (MRI) showed disc bulging at the L3/4 and L4/5 levels (Figure 1), and nerve root block injections at these levels provided marked symptom relief.

**Figure 2:**
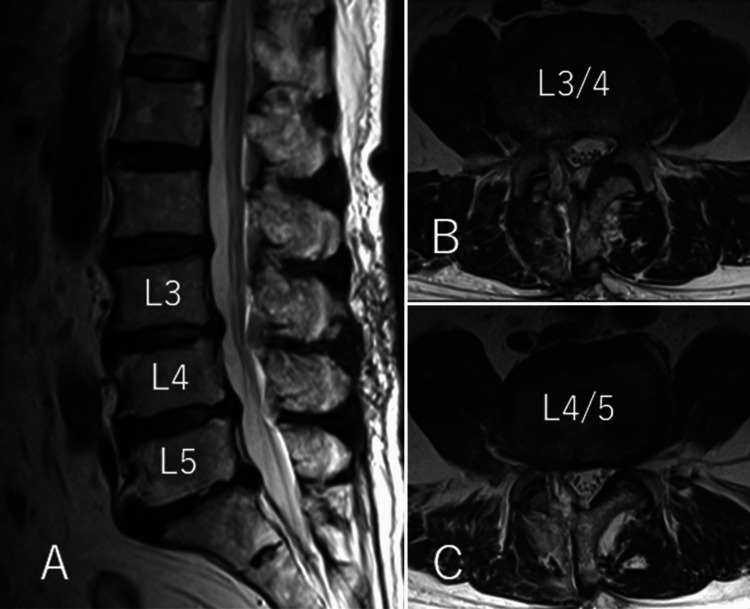
Postoperative MRI (A) Sagittal. (B,C) Axial. T2-weighted MRI performed postoperatively after 10 days showing good decompression in the spinal canal.

Based on these findings, a microscopic discectomy was performed under general anesthesia in the prone position. The patient’s body was stabilized in the prone position using a four-point support bed, which secured both sides of the thorax and the iliac regions. A midline incision was made on the back, and the right-sided paraspinal muscles were dissected. Partial laminectomies at the L3/4 and L4/5 on the right side were performed. Then, the herniated disc within the spinal canal was removed as much as possible. The surgery was completed without any evident nerve injury. Intraoperative motor evoked potentials (MEPs) showed no decrease in amplitude, and no abnormal free-running was recorded. A postoperative MRI examination performed 10 days after surgery confirmed adequate decompression at the L3/4 and L4/5 levels (Figure 2).

**Figure 3:**
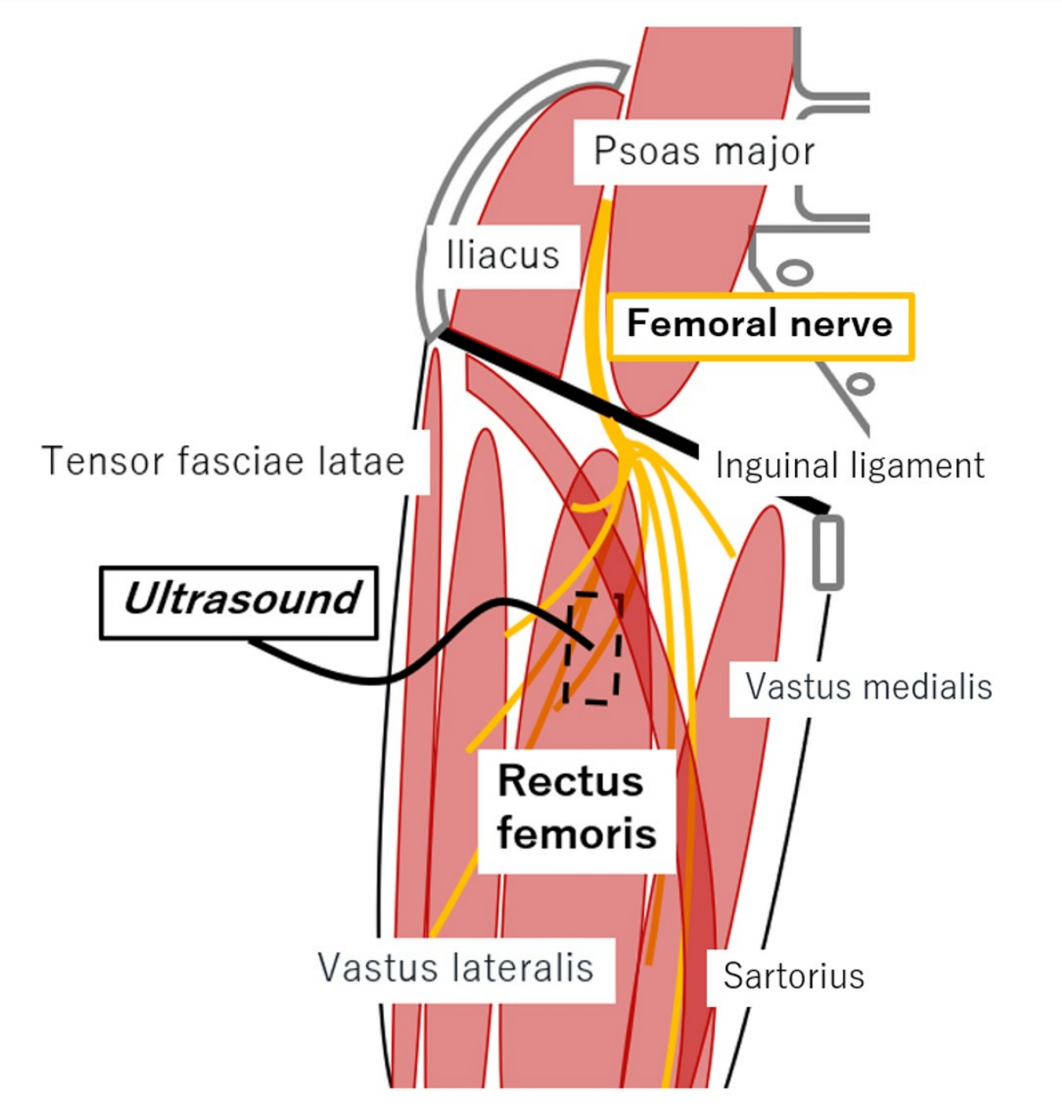
Ultrasound of the area with a positive Tinel-like sign The anatomical relationship between the muscles and the nerves in the anterior aspect of the right thigh is illustrated. The dashed rectangle indicates the position where the ultrasound probe was applied. Image credit: Original creation.

Immediately after the surgery, while the lateral thigh pain improved, the patient subsequently developed anterior thigh pain and muscle weakness, which led to difficulty walking. Manual muscle testing revealed a strength of 4/5 in hip flexion and knee extension muscles. In contrast, the knee flexors, ankle flexion and extension, and extensor hallucis longus were graded as 5/5. The pain in the front of the thigh extended from the groin to a wide area above the knee. Pain in the groin area was elicited during hip extension, and a positive Tinel-like sign was observed over the rectus femoris near the inguinal ligament. We applied ultrasound along the longitudinal axis of the rectus femoris muscle to observe the targeted structures (Figure 3).

**Figure 4:**
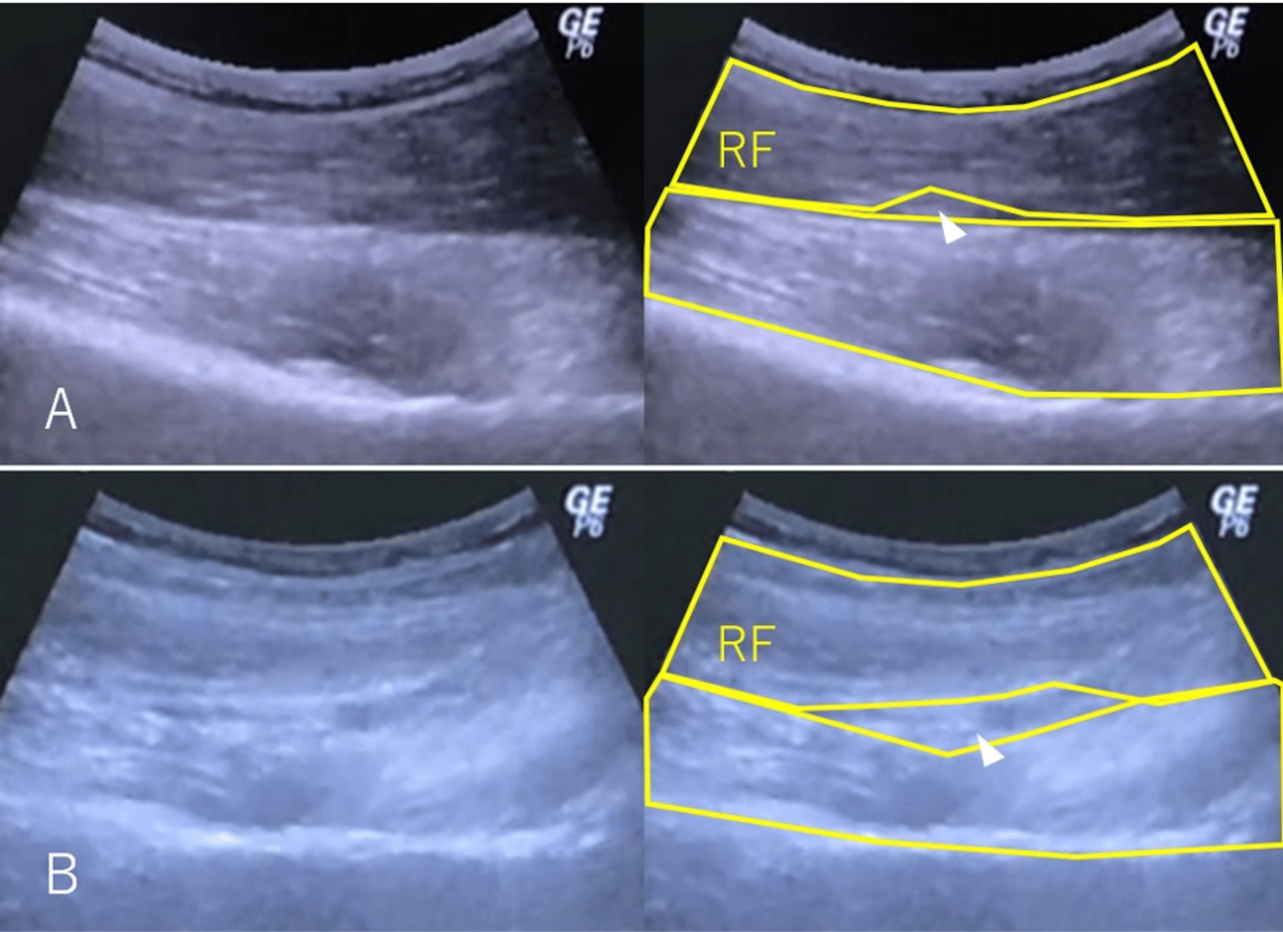
Ultrasonography conducted above the rectus femoris (RF) at the site exhibiting a positive Tinel-like sign During severe pain (A), the gap (indicated by a white arrowhead) through which the femoral nerve passed was observed. Upon symptom improvement (B), this gap was enlarged, and nerve gliding was notably enhanced.

The nerve located lateral to the femoral artery and vein was visualized as a uniform, hypoechoic structure. To evaluate the degree of femoral nerve gliding and compression, flexion and extension movements were performed. Specifically, with the hip joint extended, passive flexion and extension of the knee were conducted while simultaneously using ultrasound to observe the branches of the femoral nerve coursing beneath the rectus femoris muscle. Ultrasound imaging showed no hematoma in the rectus femoris, suggesting that nerve traction or direct compression was the likely cause of the patient's symptoms (Figure 4A). Rehabilitation focusing on promoting contraction of the rectus femoris was initiated. After two weeks of therapy, the patient significantly improved pain, muscle strength, and walking ability. The Tinel-like sign in the groin had disappeared. Ultrasound examination showed improved gliding of the femoral nerve and rectus femoris muscle, and improvement in compression of the femoral nerve in the hip extension position was confirmed (Figure 4B).

## Discussion

This case report highlights femoral nerve injury occurring during prone-position surgery. Postoperatively, the patient presented with symptoms resembling L3 or L4 radiculopathy. While the possibility of nerve injury due to surgical manipulation or microthrombi cannot be entirely excluded, intraoperative surgical findings, MEP monitoring, postoperative imaging, and symptoms did not support L3 or L4 nerve root impairment. Conversely, the broad anterior thigh pain, a positive Tinel-like sign in the groin, and diagnostic findings from postoperative MRI and ultrasonography strongly suggest that the diagnosis of femoral nerve injury is more reasonable. Potential causes of femoral nerve injury include prolonged compression or traction, inadvertent injury during surgical approaches or instrumentation, and hematoma formation in the rectus femoris [1]. Ultrasound imaging and MRI were instrumental in ruling out other causes. Given the distance between the surgical site and the femoral nerve, the injury was attributed to compression or traction due to positioning. This diagnosis enabled early initiation of rehabilitation, facilitating recovery.

In prone spinal surgery, lateral femoral cutaneous nerve injury is a relatively common complication, reported in 12%-20% of cases [9,10]. This condition is primarily attributed to direct compression, with some authors suggesting an increased risk in obese patients, while others report a high risk in thinner individuals [11]. Proper padding placement is considered a key measure to reduce this risk. In contrast, reports of femoral nerve injury during prone spinal surgery, leading to muscle weakness and sensory deficits, are rare, and the exact incidence remains unclear [8,10]. Unlike the lateral femoral cutaneous nerve, the femoral nerve lies deeper and is less likely to be affected by simple compression due to positioning. Femoral nerve injury is a recognized complication of other various procedures, including psoas abscess drainage, pelvic surgeries, and total hip arthroplasty involving retractors [1]. The prognosis of peripheral nerve injuries caused by surgical positioning is generally favorable, as these injuries often involve transient conduction block due to localized myelin damage without structural axonal injury. Recovery typically occurs within weeks to months [12]. In the present case, the patient showed improvement within two weeks.

MEPs have been reported as a valuable option for preventing femoral nerve injury. In a reported case involving anterior and posterior surgeries with fusion from L4 to S1, intraoperative MEP signals from the left quadriceps and gluteus medius disappeared but reappeared following the release of retractors [2]. Similarly, during prone spinal surgery, which is away from the femoral nerve, a reduction in MEP amplitudes from the quadriceps or gluteus muscles should prompt considerations for adjustments, such as revising hip extension or alleviating compressive forces, to avert further nerve damage. However, in the present case, no significant reductions in MEP amplitudes were recorded. This observation suggests that, unlike retractor-induced compression, the sensitivity of MEP monitoring for detecting position-related nerve injury might be relatively limited in such scenarios.

Diagnosing entrapment neuropathy without a clear compression site is challenging. In this case, ultrasound imaging confirmed the absence of hematoma in the thigh. Over time, improved visualization of the femoral nerve and enhanced muscle mobility and gliding were observed, correlating with symptom relief. In carpal tunnel syndrome, reduced nerve gliding on ultrasound has been reported as a useful diagnostic indicator for entrapment neuropathy [13]. Similarly, in this case, such findings were valuable for both diagnosis and evaluating treatment effectiveness. However, ultrasound assessments lack objectivity and currently serve only as a supplemental diagnostic tool. Future studies with more cases are needed to establish objective evaluation criteria.

## Conclusions

This case highlights the importance of recognizing femoral nerve injuries during prone-position spinal surgeries. To avoid femoral nerve injury during prone spinal surgery, it is important to prevent excessive extension and pressure on the groin and in front of the thigh. Intraoperatively, a decrease in MEP amplitudes in the affected region warrants immediate evaluation of patient positioning. Postoperatively, it is crucial to differentiate femoral nerve injury from L3 or L4 nerve root pathology, with the Tinel-like sign serving as a valuable diagnostic indicator. In addition, ultrasound imaging proved valuable for confirming the diagnosis and monitoring recovery, suggesting its potential for managing similar entrapment neuropathies. Further studies are needed to establish objective evaluation criteria and improve preventive strategies.
